# MicroRNA-Target Interaction Regulatory Network in Alzheimer’s Disease

**DOI:** 10.3390/jpm11121275

**Published:** 2021-12-02

**Authors:** Aleksander Turk, Tanja Kunej, Borut Peterlin

**Affiliations:** 1 Department of Animal Science, Biotechnical Faculty, University of Ljubljana, 1230 Domžale, Slovenia; turk.aleksander@gmail.com; 2 Clinical Institute of Genomic Medicine, University Medical Centre Ljubljana, 1000 Ljubljana, Slovenia

**Keywords:** Alzheimer’s disease, protein–protein interaction (PPI), biomarker, microRNA (miRNA), miRNA–target interaction (MTI)

## Abstract

Alzheimer’s Disease (AD) is a progressive neurodegenerative disorder and the most common cause of dementia; however, early diagnosis of the disease is challenging. Research suggests that biomarkers found in blood, such as microRNAs (miRNA), may be promising for AD diagnostics. Experimental data on miRNA–target interactions (MTI) associated with AD are scattered across databases and publications, thus making the identification of promising miRNA biomarkers for AD difficult. In response to this, a list of experimentally validated AD-associated MTIs was obtained from miRTarBase. Cytoscape was used to create a visual MTI network. STRING software was used for protein–protein interaction analysis and mirPath was used for pathway enrichment analysis. Several targets regulated by multiple miRNAs were identified, including: *BACE1*, *APP*, *NCSTN*, *SP1*, *SIRT1*, and *PTEN*. The miRNA with the highest numbers of interactions in the network were: miR-9, miR-16, miR-34a, miR-106a, miR-107, miR-125b, miR-146, and miR-181c. The analysis revealed seven subnetworks, representing disease modules which have a potential for further biomarker development. The obtained MTI network is not yet complete, and additional studies are needed for the comprehensive understanding of the AD-associated miRNA targetome.

## 1. Introduction

Alzheimer’s disease (AD) is a complex, multifactorial, progressive neurodegenerative disorder afflicting the central nervous system (CNS) and is the most common cause of dementia. The disease’s clinical progression is variable with several contributing factors, is irreversible and inevitably fatal [1]. The cause of the disease is mostly still unknown. It has been associated with the accumulation of misfolded amyloid beta (Aβ) proteins, hyperphosphorylation of tau proteins, inflammation, the formation of neurofibrillary tangles, and single-nucleotide polymorphisms (SNPs) in certain AD-associated genes, such as the *APOE* gene [2]. AD is characterized by the loss of neurons and synapses in the brain, leading to a gradual loss of cognitive function. Disease progression is divided into three clinical stages: preclinical, prodromal, and dementia stages. In the disease’s early stages, this manifests through episodes of forgetfulness, such as forgetting the names of family members and friends and confusion in unfamiliar situations. As the disease progresses, more regions of the brain are affected, resulting in severe difficulties with speech, thought, motor control, and other functions. Late-stage AD outcomes include irreversible disruptions to visual and visuospatial perception, behavioral alterations, losing one’s ability to care for oneself, and progressively worsening cognitive and memory faculties. The formation of new memories becomes highly impaired, though older memories are often retained [1]. In 2015, it was estimated that 29.8 million people worldwide were living with AD [3].

Diagnosing AD is often carried out by interviewing relatives about the person’s overall health, medical history, drug use, and other relevant information. Cognitive tests can also be performed along with blood and urine tests. Brain scans may be used to rule out other causes of dementia; these include computed tomography (CT), magnetic resonance imaging (MRI), and positron emission tomography (PET) scans. Modern diagnostic methods are based on IWG-2 criteria, which rely on both biomarker and clinical phenotypes [4]. Despite these diagnostic methods, a definitive diagnosis can only be made after death with the examination of brain tissue. This is changing, however, as advancements in biomarker research are allowing more accurate assessment of the presence of AD. Three biomarkers have been established and examined in depth: Aβ proteins, tau protein, and phosphorylated tau proteins. The current AD biomarker panel is categorized into three types of biomarker evidence for pathology, known together as the ATN classification system. This system allows individuals to be analyzed for three parameters: alterations of Aβ proteins (in CSF or detected with PET scans; A), the hyperphosphorylation of tau proteins (in CSF or PET scans; T), and neurodegeneration levels (PET, MRI, and others; N) [5]. Accumulating evidence suggests that biomarkers found in blood (circulatory biomarkers) may be promising in identifying cases of AD. These include microRNAs (miRNAs), inflammatory markers, blood-based Aβ markers, and biomarkers for oxidative stress [2].

In recent years, progress has been made in the field of biomarker development for AD. Analyzing Aβ proteins in plasma together with other blood biomarkers can accurately detect cerebral Aβ. This method yields even more accurate results when combined with *APOE* genotyping and thus reduces the cost of PET scans and need for lumbar punctures [6]. CSF and plasma p-tau181 and p-tau217 levels have also been studied as potential early biomarkers of AD [7]. These biomarkers were identified to be present in significantly higher concentrations in patients with early or mild AD. PET scans for tau proteins, on the other hand, presented more accurate results when gauging disease progression [8]. It has also been shown that plasma biomarkers, such as p-tau217, can be used to reduce the necessary sample size for future clinical trials [9]. Blood assay for p-tau181 has been identified as a promising biomarker for AD pathology. P-tau 181 has also been identified as a promising AD biomarker and has been shown to provide high diagnostic accuracy and the ability to differentiate AD from several other neurodegenerative diseases [10].

miRNAs are a class of short, non-coding RNA consisting of about 22 nucleotides. They play a role in the post-transcriptional regulation of gene expression as they, along with their associated proteins, bind to mRNA. This protein–miRNA complex then mediates either the degradation, destabilization, or repression of the mRNA. miRNA have been shown to have a wide array of targets, and one miRNA can target multiple mRNAs. It has also been shown that over 60% of protein-coding genes in humans have been under selective pressure to maintain sequences that would allow miRNA binding. Over 2000 human miRNA have so far been identified, and they are involved in numerous physiological processes as well as disease development [11,12,13].

Multiple miRNAs have thus far been associated with AD [2,14]. These miRNAs were identified as downregulated or upregulated in AD patients compared to healthy controls, and some have been shown to regulate AD-associated genes, such as *APP* and tau protein genes [14]. Plasma concentrations of miR-15b and miR-125b have been proposed as biomarkers of AD pathophysiology, and the study proposes a pathway-based approach to therapies for AD [15]. miRNA expression in AD is, similar to other contributing factors to the disease, highly heterogenous. A precision medicine approach to AD diagnostics has been proposed that includes: biomarker testing, and PET and MRI scans during the prodromal period of AD. This approach would allow more accurate disease trajectory predictions and treatment based on the individual patient’s genetic, epigenetic, and neuroimaging profile [1]. miRNAs are also involved in both of the leading hypotheses for AD development—the amyloid cascade hypothesis and the tau hypothesis [16]. Nineteen miRNAs have been identified as having diagnostic potential in human AD studies. Among them, miR-206, miR-181a, and miR-146a from blood samples have shown the ability to predict whether mild cognitive impairment would progress to AD [17,18]. Anti-miRNA (AM) treatments have also been proposed- using miRNA complementary strands of RNA. The AM approach has shown promise in murine models and cell cultures [19,20]. AM strategies for AD patients will likely require a more individual-focused approach to disease treatment, tailored to the individual’s needs due to AD heterogeneity [21]. Potential therapeutic approaches are, however, not limited to AM strategies. Studies on cell cultures and animal models have also identified compounds that affect miRNA expression [22]. Current commonly used diagnostic methods are primarily based on CSF biomarkers. As circulatory miRNAs can be assessed in blood and do not require a lumbar puncture, their usage as biomarkers for diagnosis or potential treatment may be advantageous. While miRNAs may prove to be the preferable AD biomarker, data on their interactions with targets are fragmented, making it difficult for researchers to find a comprehensive overview of MTIs.

The aim of the present study was to: 1. review published data on the currently known miRNAs associated with AD, 2. present this information as the miRNA–target network to identify central molecules with potential for biomarker and therapeutic target development, and 3. conduct a pathway enrichment analysis and protein interaction analysis.

## 2. Methods

AD-associated miRNAs were retrieved from the online database miRTarBase, release 7.0 (http://mirtarbase.mbc.nctu.edu.tw/php/index.php) (accessed on 21 September 2021) [23]. The database contains experimentally validated MTIs. These MTIs were validated by using various methodologies, including reporter assay, Western blot, quantitative PCR (qPCR), microarrays, next-generation sequencing (NGS), and pSILAC. All data obtained from the database were manually reviewed. Each entry was also manually verified and only MTIs reported to be associated with AD were included in the analysis. The visual representation of the MTI network was made using the Cytoscape tool (https://cytoscape.org) (accessed on 24 September 2021) [24]. miRNA target genes were investigated for known protein–protein interactions (PPI) using the STRING database (https://string-db.org/) (accessed on 24 September 2021) [25]. AD-associated miRNAs were also analyzed for enrichment in biological pathways using mirPath v.3 (http://diana.imis.athena-innovation.gr/DianaTools/index.php) (accessed on 2 November 2021) [26]. MirPath is a prediction-based bioinformatics tool that enables the identification of biological pathways in which the query miRNAs’ target genes are enriched. The MirPath’s KEGG analysis tool was utilized using the following parameters: *p*-value threshold of 0.05 and conservative estimates applied to the MicroT-CDS search algorithm. The obtained enriched biological pathways were manually reviewed for association with AD in previously published literature.

## 3. Results

**Figure 1:**
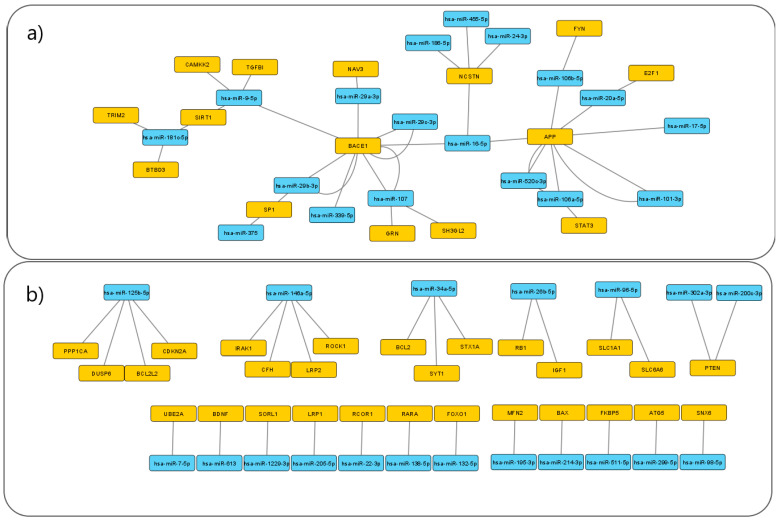
Network of experimentally validated MTIs associated with AD visualized using the Cytoscape software. Orange-colored nodes represent target genes while blue-colored nodes represent miRNAs. Each edge represents an experimentally validated interaction between a miRNA and its target. (**a**) The largest subnetwork identified in the data set, consisting of 18 miRNAs and 15 target genes, with a total of 37 MTIs. (**b**) Smaller subnetworks and miRNA–target pairs identified in the data set.

A total of 37 unique miRNAs associated with AD were extracted from miRTarBase. The network consists of 37 miRNAs and 43 target genes, which are connected through 66 MTIs and experimentally validated by 45 unique articles. A list of MTIs is presented in Table 1 [27,28,29,30,31,32,33,34,35,36,37,38,39,40,41,42,43,44,45,46,47,48,49,50,51,52,53,54,55,56,57,58,59,60,61,62,63,64,65,66,67,68,69,70,71]. All MTIs presented in these results have been previously experimentally validated. The MTI network was visualized using Cytoscape software and is shown in Figure 1. miR-9, miR-107, miR-125b, and miR-146a were among miRNAs with the highest number of interactions, with four MTIs each. miR-16, miR-34a, miR-106a, and miR-181c were also highly connected, with three MTIs each. The most prominent miRNA targets were *BACE1*, *APP*, and *NCSTN*, which were the target of seven, seven, and four miRNAs, respectively. miRNAs and targets connected by multiple edges represent interactions confirmed by multiple independent experiments, such as the connections between *BACE1* and hsa-miR-107. The results revealed a larger subnetwork consisting of 18 miRNA and 15 targets. Additionally, six smaller subnetworks of up to four MTIs were also identified. Twelve reported MTIs were between pairs of a single miRNA and target and were not part of a larger network. The targets of these 12 MTIs were: *ATG5*, *BAX*, *BDNF*, *FKBP5*, *FOXO1*, *LRP1*, *MFN2*, *RARA*, *RCOR1*, *SNX6*, *SORL1*, and *UBE2A* (Figure 1). The complete table of MTIs, which includes miRTarBase IDs and experimental validation methods for each MTI, is available in Supplementary Data (Supplementary Table S1).

**Figure 2:**
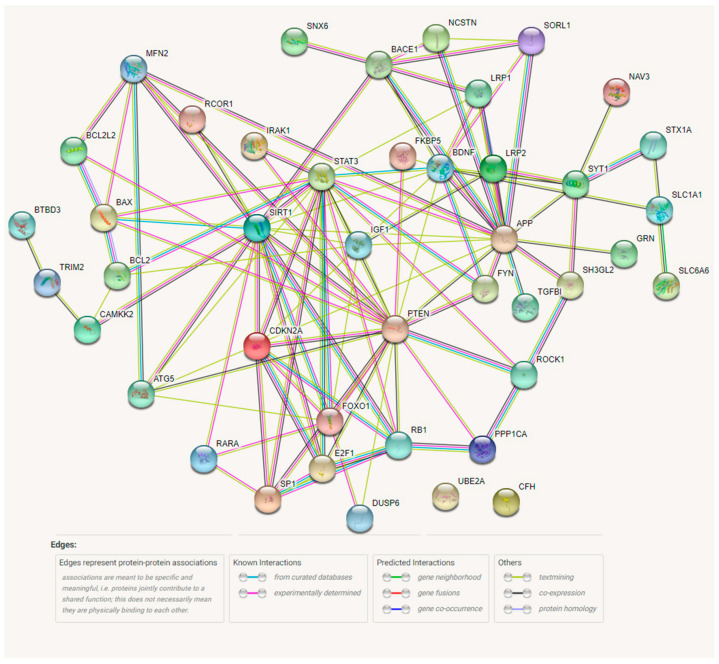
Protein–protein interaction network of 43 AD-associated miRNA targets using STRING software. The colors of connections between nodes represent the type interaction between the two proteins. The most reliable of these connections are “known interactions” from curated databases and experiments. Common gene neighborhoods, fusions, and co-occurrences as well as textmining, co-expression, and protein homology are considered less reliable connections and tend to require independent verification.

Target genes (*n* = 43) were then explored for PPIs using the STRING tool (Figure 2). A total of 41 of the 43 targets were part of a large PPI network; only two targets, *UBE2A* and *CFH*, had no known interactions with the rest of the proteins in the network. The network includes 43 nodes with 110 interactions, which is significantly more interactions than expected (PPI enrichment *p*-value: <1.0^−16^). Some proteins had notably more interactions than others, representing hubs. The five proteins with the most PPIs were: PTEN, SIRT1, APP, STAT3, BACE1, with 19, 16, 16, 9, and 7 PPIs, respectively. These five proteins are therefore present in 67 of the 110 PPIs in the network, thus representing central hub proteins.

To identify pathways in which 37 AD-associated miRNAs were enriched, we conducted an analysis with the mirPath tool. The analysis conducted using mirPath identified enrichments of the 37 unique AD-associated miRNAs in 68 biological pathways. Biological pathways were manually reviewed for association with AD based on literature. A total of 44 of the 68 biological pathways were associated with AD in previously published literature (Table 2) [69,70,71,72,73,74,75,76,77,78,79,80,81,82,83,84,85,86,87,88,89,90,91,92,93,94,95,96,97,98,99,100,101,102,103,104,105,106,107,108,109,110,111,112]. Thus, a total of 87 articles were reviewed for MTIs and enriched biological pathways. Supplementary Data (Supplementary Table S2) includes the full results of the pathway enrichment analysis.

## 4. Discussion

In the present study, we formed an MTI network based on data on AD-associated miRNA, extracted from miRTarBase. This network contained seven MTI subnetworks and 12 MTI pairs. Nodes in the network with the highest number of edges include *APP*, *BACE1*, *NCSTN*, *SIRT*, and *SP1*, as well as the following miRNAs: miR-9, miR-16, miR-34a, miR-106a, miR-107, miR-125b, miR-146, and miR-181c. We also investigated the miRNA targets for their interactions with other proteins, and visualized a PPI network, using STRING software. Furthermore, we conducted an enrichment analysis for AD-associated miRNAs.

Previously published literature indicates that miRNAs are an important regulatory mechanism for AD-associated gene expression [14]. So far, several miRNAs have been shown to regulate AD-associated genes [14]. As the main miRNA mechanism of action is the downregulation of target genes, it is important to assess whether they are being over- or under-expressed in patients. Additionally, miRNA expression can be tissue-specific or bound to a specific mechanism, such as the regulation of extracellular vesicles, which are involved in cell communication [116]. As the understanding of the AD genetic background is not yet complete, observing miRNAs as a contributing factor may prove valuable.

The methods used for the validation of miRNA–target interactions are not all equally reliable and have different validation statuses. The miRTarBase methods are divided into strong and less strong based on the validation status. Methods such as Western blot, qPCR, and reporter assay are considered to give more reliable information and are marked as methods with strong validation status. Microarrays, NGS, pSILAC, and other methods are, by contrast, considered to generate less strong evidence. Consensus on the validation strength of methods has not yet been achieved as studies use different definitions of what constitutes strong and less strong evidence when it comes to MTIs. The edges between targets and miRNAs in Figure 1 do not distinguish between strong and less strong validation. In the future, these data could be accounted for in the graphical network.

The study results identified MTI subnetworks of varying sizes. The largest MTI subnetwork identified consists of 18 miRNAs and 15 target genes. The most prominent miRNA targets in the MTI network are *APP*, *BACE1*, *NCSTN*, *SIRT*, and *SP1* as they are the targets of multiple miRNAs. The six smaller networks are composed of three to five nodes with two to four MTIs. In one subnetwork, two miRNAs regulate the same gene; where hsa-miR-302a-3p and hsa-miR-200c-3p both target *PTEN*. From Figure 1, it is also apparent that there are 12 MTI pairs not connected to the other subnetworks.

As previously mentioned, five genes had the largest number of edges in the MTI network. Among these is the *BACE1* gene, regulated by seven AD-associated miRNAs in the MTI network. Beta-secretase 1 is a protease encoded by the *BACE1* gene. Its main role is the extracellular cleavage of the amyloid precursor protein (APP). It cleaves APP into two components, one of which is known as C99. This component is then further cleaved by γ-secretase, releasing an amyloid beta peptide (Aβ), which is the primary component in amyloid plaques. These plaques are commonly found in the brains of AD patients. Due to the correlation of amyloid plaque formation and AD, BACE1 has been closely studied. Inhibiting *BACE1* would prevent the formation of Aβ, and it has been speculated that *BACE1* inhibitors may prevent the development of the disease [117].

Another miRNA target with multiple MTIs is *APP*. Like *BACE1*, *APP* is also regulated by seven miRNAs in the MTI network. As seen in Figure 1, *BACE1* and *APP* are both targeted by miR-16. As previously mentioned, APP is cleaved by proteases and is the precursor for Aβ. Despite being of great interest in connection to AD, its function is not completely known. Evidence shows that increased *APP* expression could promote the production of Aβ, leading to a negative impact on neurons and synapses [118].

Nicastrin is a protein encoded by the *NCSTN* gene. *NCSTN* is targeted by four miRNAs in the MTI network, making it the gene with the third-highest number of interactions with AD-associated miRNA. It is part of the γ-secretase protein complex and is thus connected to the formation of Aβ. No solid evidence has so far been found that would connect it to the development of AD, though this does not exclude it as a potential contributing factor [119]. As seen in Figure 1, miR-16 regulates three highly interconnected genes in the MTI network—*APP*, *BACE1*, and *NCSTN*.

The *SIRT1* gene encodes the enzyme NAD-dependent protein deacetylase sirtuin-1 (SIRT1). In the MTI network, *SIRT1* is a target for two miRNAs: miR-181c and miR-9.

The roles and functions of human sirtuins are still largely unknown; however, SIRT1 has a known interaction with hypoxia-inducible factors 1α and 2α (HIF1A and EPAS1, respectively). HIF-1α and EPAS1 are important for proper brain development as they are crucial for cell adaptation to hypoxia [120]. In murine models, all gene expression alterations in *EPAS1*-deficient mice have previously been associated with AD and memory loss [121]. SIRT1 was found to deacetylate the tau protein in some cell cultures. Among other interactions, it was also observed that it has a protective role in microglia-dependent Aβ toxicity [122].

SP1 is a transcription factor and is targeted by two miRNAs in the MTI network: miR-29b and miR-375. *SP1* may be involved in the development of AD as it can regulate the expression of several genes previously associated with AD, such as *APP* and tau protein genes. *SP1* has also been shown to be significantly upregulated in the frontal cortex of AD patients [123].

*PTEN* is a gene that translates into the phosphatase and tensin homolog (PTEN) protein. *PTEN* is a target of miR-200c and miR-302a in the MTI network. Mutations in this gene are primarily associated with different types of cancer; however, they are also associated with AD through its role in synaptic and cognitive functions [124]. The gene also acts as a tumor suppressor, and its involvement with AD has been studied in mice [125].

Along with an MTI network, we also conducted a PPI analysis for AD-associated miRNA targets (Figure 2). Both the PPI and MTI network contained nodes with a large number of interactions, either with other targets (proteins) or miRNAs. The two networks share some nodes with multiple interactions. *APP*, *BACE1*, *PTEN*, *SIRT1*, and *SP1* are targets that have the highest number of interactions in both networks. However, the PPI networks also include other highly connected proteins, such as STAT3, CDKN2A, E2F1, MFN2, RB1, and IGF1. The PPI network is highly interconnected: 41 of the 43 genes currently have at least one known or predicted interaction within the network. This level of interconnectedness of miRNA targets further points to the complex nature of AD.

A total of 37 AD-associated miRNAs were identified to be enriched in 68 biological pathways using the mirPath tool. For these 68 pathways, we conducted a secondary review of previously published literature and identified 44 pathways which have previously been described in association with AD. These 44 AD-associated pathways also include five pathways associated with other diseases: glioma, acute myeloid leukemia, colorectal cancer, hepatitis B, and type II diabetes mellitus. For example, glioma and AD share some common biological pathways associated with their development as well as genetic and environmental risk factors but are as of yet not causally related [75]. Common pathways with AD were also identified for acute myeloid leukemia [126] and colorectal cancer [80]. Interestingly, research has discovered an inverse correlation between AD risk and lung cancers [127] as well as other types of cancers [128,129,130]. Further studying the shared pathways between these diseases may yield additional insight into the role played by individual pathways in the development of AD.

Among the 44 AD-associated pathways, 39 pathways were not associated with diseases. These pathways are involved in inter- and intracellular signaling, gene regulation, cell adhesion, endocytosis, phagocytosis, and inflammation, which is expected, as these mechanisms have been shown to be involved in AD pathology [131]. The results of the analysis indicate that AD-associated miRNAs are involved in a variety of biological pathways. Based on the number and variety of pathways miRNA target genes are enriched in, miRNA appear to play a role in AD on multiple levels. Observing the disease at its endpoint, however, as is the tendency of study designs for AD-associated factors, has drawbacks. Due to the complexity of cellular regulatory mechanisms, observing dysregulations at the end point of a disease may not necessarily answer questions regarding its etiology. The interconnected nature of biological pathways means that the dysregulation of one pathway can cause the dysregulation of a second pathway. Though the second pathway is now dysregulated, studies observing the end-point of a disease’s progression will not be able to discern between the cause and effect [132]. Therefore, longitudinal studies spanning from the preclinical stage of disease development to its endpoint are vital for the understanding of AD and consequently identifying therapeutic approaches.

MTIs acquired from miRTarBase do not include all genes whose variants are commonly associated with increased risk of AD. Their absence may be due to the incomplete initial data set, the current lack of knowledge about the role of miRNA in the contribution to AD development, or an indirect mechanism through which these risk variants contribute to the disease. Some genes commonly associated with AD, such as *APOE*, are currently not included in the database. *APOE* is considered one of the most influential genetic risk factors for late-onset AD—specifically, one of its three major isoforms, APOE-E4. The full extent of interactions between *APOE* and miRNA is not yet understood; however, it has been shown that levels of miR-1908 were negatively correlated with APOE expression [133]. Other known genetic AD risk factors include *PSEN1* and *PSEN2*, specifically for early-onset AD [134]. *PSEN1* is regulated by miR-193a [135] while *PSEN2* knockout microglia cells exhibited the downregulation of miR-146 [43]. Further research into the involvement of *APOE*, *PSEN1*, and *PSEN2* is required in order to acquire a better understanding of their role in AD. Future research is needed to reveal complete understanding of the role of miRNA in *APOE*, *PSEN1*, and *PSEN2* regulation.

Different methods have been used both for the identification of novel biomarkers and for diagnostic purposes. For example, a cell culture reporter assay was used to determine that miR-107 regulates the expression of *BACE1* [57]. Cell culture reporter assays, ELISA, xMap Luminex, shotgun proteomics, and other methods are commonly used to perform biomarker assays. The methodology on AD biomarker detection, however, is not entirely consistent among laboratories. For example, individual laboratories have different concentrations of Aβ that are considered low or high for the purpose of assays [136]. Blennow and Zetterberg (2018) have evaluated a large number of studies on miRNAs associated with AD and highlighted the need for a standardized analytical protocol among research centers [2]. A standardized approach for determining whether AD-associated molecules are present in low or high concentrations for the purpose of diagnostics is necessary for reliable, reproducible studies on AD.

Despite several important contributions to the development of the study field, there are also some limitations inherent to the present study. The study is limited by data available in the miRTarBase and mirPath databases, as these databases do not include all miRNAs and their targets currently known to be associated with AD. MirTarBase is one of the most extensive miRNA databases, but due to the rapid pace of the developments in this field, it may be challenging to keep a database up to date. Additionally, our initial dataset did not include expression levels and tissue specificity for miRNA and targets. The stage of AD during which measurements were taken was also not included, though these data could be taken into account in the future. The focus of the present study is the identification of interactions between known AD-associated miRNA and targets, their visualization, and the analysis of their enrichment in biological pathways. Through this, the study contribution is an overview of the interplay between miRNAs and AD-associated genes.

miRNAs have been extensively studied for their use as AD biomarkers in previously published literature [137]. A study by Lugli et al. (2015) assessing exosomal miRNAs as potential AD biomarkers has observed the differential presence of miRNAs in the plasma of AD patients. Twenty miRNAs showed notable differences, and seven of those were used for AD status prediction of patients using machine learning. The machine learning model predicted the patient’s AD status based on samples with an 83–89% accuracy; however, the authors recommend a replication with a larger cohort. The addition of miRNA expression data into other AD biomarker diagnostic tests is likely to further increase the diagnostic accuracy [138]. The results of a study by Leidinger et al. (2013) showed that a panel of 12 blood-based miRNAs can be used to differentiate between AD patients and healthy controls with 93% accuracy. This panel of 12 miRNAs can also differentiate between AD and other CNS disorders with 74–78% accuracy [139]. Other studies testing circulatory miRNAs as biomarkers have also shown 75–95% accuracies in identifying AD [140]. These studies are, however, focused on the late stages of AD. Integrating blood or plasma miRNA biomarkers with other biomarkers, such as Aβ40 and Aβ42, are likely prospective methods of early disease detection.

In the present study, miR-9, miR-16, miR-34a, miR-106a, miR-107, miR-125b, miR-146, and miR-181c were miRNAs present in the highest number of MTIs in the network. These miRNAs present promising components of regulation of AD-associated genes. miR-16 and miR-34a are involved in processes key to the amyloid cascade hypothesis model of AD development [14]. miR-16 inhibits *APP* expression while miR-34a inhibits the expression of proteins connected with Aβ clearance [14]. miR-125b, meanwhile, is involved in the tau cascade hypothesis model, where its role is the inhibition of kinases responsible for tau hyperphosphorylation [14]. These miRNAs are differentially expressed in the brain and CSF; however, as diagnostic methods aim towards blood-based biomarkers, studies are necessary to elucidate whether they are also viable as circulatory biomarkers. Studies have shown the potential for blood-based miRNA biomarkers [137,138,139,140]. However, studies involving miRNA as circulatory biomarkers have, in the majority of cases, been performed with participants at the dementia stage of AD. Longitudinal studies with larger sample sizes are necessary to identify a combination of robust early detection biomarkers. As for therapeutic targets, research in the topic remains incomplete, being performed predominantly on cell cultures and murine models. Anti-miRNA (AM) approaches are challenged by an imperfect drug delivery system and unwanted effects on the expression of non-AD-associated genes, due to the multi-target nature of miRNAs. An anti-miR-146a (AM-146a) approach has shown a regained miRNA-associated homeostasis in murine models of AD. In cell cultures, AM-34a has returned overexpressed *TREM2* [141] and *SHANK3* [21] levels back to expected, normal levels, and thus, homeostasis. miRNAs as therapeutic targets should therefore not be ruled out, but more research is necessary to identify their level of potential for this purpose.

miRNAs play a prominent role in the regulation of AD-associated gene expression, with vast research potential into targets for screening, diagnosis, or treatment. Our analysis revealed seven subnetworks of MTIs, representing disease modules, which have the potential for network-based biomarker development. Further investigation into the cause of the upregulation or downregulation of miRNA may also prove useful in the search for the cause of AD. As there are a large number of miRNAs to consider in AD development, research, or screening, lab-on-a-chip technology is likely to be an efficient and cost-effective method to utilize.

## 5. Conclusions

In the present study, we conducted a synthesis of heterogenous results extracted from 88 unique studies; MTIs were obtained from 45 articles and AD-associated pathways from 43 articles. MTI data were visualized in the form of a network. A visual representation of experimentally validated MTIs has revealed potential novel network-based biomarkers. The miRNAs, their targets, MTIs, and associated biological pathways identified in this study hold potential for understanding AD progression. They also hold potential for additional circulatory biomarker development and therapeutic targets, as they are involved in multiple key molecules and mechanisms associated with AD. As research in the field grows, it is becoming more apparent that the role of miRNA in the development of AD is substantial and holds potential for the development of improved future diagnostics and therapeutic approaches. The results identified miRNAs and target genes representing central molecules, which will enable the formation of new hypotheses in the future. Research involving longitudinal studies and incorporating both miRNAs and other known biomarkers would allow the development of a more complete view of AD for advancements in disease screening, diagnosis, and treatment.

## Figures and Tables

**Table 1:** Experimentally validated MTIs associated with AD obtained from miRTarBase and literature [27,28,29,30,31,32,33,34,35,36,37,38,39,40,41,42,43,44,45,46,47,48,49,50,51,52,53,54,55,56,57,58,59,60,61,62,63,64,65,66,67,68,69,70,71].

miRNA	Target Gene Symbol	Target Gene (Entrez Gene ID)	Reference (PMID)
hsa-miR-20a-5p	*E2F1*	1869	19110058 [39]
hsa-miR-146a-5p	*CFH*	3075	18801740 [51]
hsa-miR-106b-5p	*APP*	351	19110058 [39]
hsa-miR-101-3p	*APP*	351	20395292 [59]
hsa-miR-101-3p	*APP*	351	21172309 [67]
hsa-miR-29b-3p	*SP1*	6667	23435408 [32]
hsa-miR-146a-5p	*IRAK1*	3654	23952003 [43]
hsa-miR-107	*BACE1*	23621	18234899 [57]
hsa-miR-107	*BACE1*	23621	20489155 [42]
hsa-miR-29b-3p	*BACE1*	23621	18434550 [60]
hsa-miR-29b-3p	*BACE1*	23621	26818210 [62]
hsa-miR-146a-5p	*ROCK1*	6093	27221467 [48]
hsa-miR-205-5p	*LRP1*	4035	19665999 [40]
hsa-miR-9-5p	*BACE1*	23621	18434550 [60]
hsa-miR-29a-3p	*BACE1*	23621	18434550 [60]
hsa-miR-520c-3p	*APP*	351	18684319 [63]
hsa-miR-106a-5p	*APP*	351	19110058 [39]
hsa-miR-106a-5p	*APP*	351	18684319 [63]
hsa-miR-34a-5p	*BCL2*	596	19683563 [49]
hsa-miR-20a-5p	*APP*	351	19110058 [39]
hsa-miR-17-5p	*APP*	351	19110058 [39]
hsa-miR-125b-5p	*CDKN2A*	1029	20347935 [50]
hsa-miR-107	*GRN*	2896	20489155 [42]
hsa-miR-125b-5p	*PPP1CA*	5499	25001178 [34]
hsa-miR-34a-5p	*SYT1*	6857	22160687 [54]
hsa-miR-34a-5p	*STX1A*	6804	22160687 [54]
hsa-miR-29a-3p	*NAV3*	89795	20202123 [27]
hsa-miR-375	*SP1*	6667	23435408 [32]
hsa-miR-181c-5p	*TRIM2*	23321	21720722 [37]
hsa-miR-181c-5p	*SIRT1*	23411	21720722 [37]
hsa-miR-9-5p	*SIRT1*	23411	21720722 [37]
hsa-miR-9-5p	*TGFBI*	7045	21720722 [37]
hsa-miR-181c-5p	*BTBD3*	22903	21720722 [37]
hsa-miR-22-3p	*RCOR1*	23186	23349832 [47]
hsa-miR-29c-3p	*BACE1*	23621	21565331 [70]
hsa-miR-29c-3p	*BACE1*	23621	25973041 [29]
hsa-miR-16-5p	*APP*	351	26440600 [46]
hsa-miR-138-5p	*RARA*	5914	25680531 [64]
hsa-miR-125b-5p	*BCL2L2*	599	25001178 [34]
hsa-miR-106a-5p	*STAT3*	6774	23399684 [31]
hsa-miR-132-5p	*FOXO1*	2308	24014289 [53]
hsa-miR-96-5p	*SLC1A1*	6505	24304186 [56]
hsa-miR-96-5p	*SLC6A6*	6533	24304186 [56]
hsa-miR-195-3p	*MFN2*	9927	27693395 [71]
hsa-miR-26b-5p	*RB1*	5925	24027266 [33]
hsa-miR-339-5p	*BACE1*	23621	24352696 [69]
hsa-miR-214-3p	*BAX*	581	23408966 [35]
hsa-miR-455-5p	*NCSTN*	23385	25100943 [30]
hsa-miR-186-5p	*NCSTN*	23385	25100943 [30]
hsa-miR-24-3p	*NCSTN*	23385	25100943 [30]
hsa-miR-125b-5p	*DUSP6*	1848	25001178 [34]
hsa-miR-107	*SH3GL2*	6456	27038654 [65]
hsa-miR-511-5p	*FKBP5*	2289	27334923 [66]
hsa-miR-299-5p	*ATG5*	9474	27080144 [52]
hsa-miR-98-5p	*SNX6*	58533	27541017 [28]
hsa-miR-16-5p	*BACE1*	23621	26440600 [46]
hsa-miR-16-5p	*NCSTN*	23385	26440600 [46]
hsa-miR-106b-5p	*FYN*	2534	27520374 [58]
hsa-miR-26b-5p	*IGF1*	3479	26847596 [55]
hsa-miR-302a-3p	*PTEN*	5728	26890744 [41]
hsa-miR-9-5p	*CAMKK2*	10645	27394443 [45]
hsa-miR-200c-3p	*PTEN*	5728	28008308 [68]
hsa-miR-146a-5p	*LRP2*	4036	27241555 [38]
hsa-miR-7-5p	*UBE2A*	7319	27929395 [61]
hsa-miR-613	*BDNF*	627	27545218 [44]
hsa-miR-1229-3p	*SORL1*	6653	27328823 [36]

Legend: *APP*: amyloid beta precursor protein, *ATG5*: autophagy related 5, *BACE1*: beta-secretase 1, *BAX*: BCL2 associated X, apoptosis regulator, *BCL2*: BCL2 apoptosis regulator, *BCL2L2*: BCL2 like 2, *BDNF*: brain derived neurotrophic factor, *BTBD3*: BTB domain containing 3, *CAMKK2*: calcium/calmodulin dependent protein kinase 2, *CDKN2A*: cyclin dependent kinase inhibitor 2A, *CFH*: complement factor H, *DUSP6*: dual specificity phosphatase 6, *E2F1*: E2F transcription factor 2, *FKBP5*: FKBP prolyl isomerase 5, *FOXO1*: forkhead box O1, *FYN*: FYN proto-oncogene, Src family tyrosine kinase, *GRN*: granulin precursor. *IGF1*: insulin like growth factor 1. *IRAK1*: interleukin 1 receptor associated kinase 1. *LRP1*: LDL receptor related protein 1, *LRP2*: LDL receptor related protein 2, *MFN2*: mitofusin 2, *NAV3*: neuron navigator 3, *NCSTN*: nicastrin, *PPP1CA*: protein phosphatase 1 catalytic subunit alpha, *PTEN*: phosphatase and tensin homolog, *RARA*: retinoic acid receptor alpha, *RB1*: RB transcriptional corepressor 1, *RCOR1*: REST corepressor 1, *ROCK1*: Rho associated coiled-coil containing protein kinase 1, *SH3GL2*: SH3 domain containing GRB2 like 2, endophilin A1, *SIRT1*: sirtuin 1, *SLC1A1*: solute carrier family 1 member 1, *SLC6A6*: solute carrier family 6 member 6, *SNX6*: sorting nexin 6, *SORL1*: sortilin related receptor 1, *SP1*: Sp1 transcription factor, *STAT3*: signal transducer and activator of transcription 3, *STX1A*; syntaxin 1A, *SYT1*: synaptotagmin 1, *TGFBI*: transforming growth factor beta induced, *TRIM2*: tripartite motif containing 2, *UBE2A*: ubiquitin conjugating enzyme E2 A.

**Table 2:** Results of the pathway enrichment analysis using mirPath tool. The table includes pathways associated with AD in previously published literature. A total of 37 AD-associated miRNAs were enriched in 68 pathways. This table includes 44 pathways, which were associated with AD in previously published literature. The PMID (Reference) column includes references to publications associating the KEGG pathway with AD [72,73,74,75,76,77,78,79,80,81,82,83,84,85,86,87,88,89,90,91,92,93,94,95,96,97,98,99,100,101,102,103,104,105,106,107,108,109,110,111,112,113,114,115].

KEGG Pathway	*p*-Value	Number of Genes	Number of miRNAs	PMID (Reference)
Axon guidance	2.03 × 10^−8^	94	35	33675023 [103]
Glioma	2.38 × 10^−8^	51	36	30560246 [75]
ErbB signaling pathway	3.03 × 10^−8^	71	37	21829755 [96]
Adherens junction	3.23 × 10^−8^	61	35	27141420 [78]
Hippo signaling pathway	5.39 × 10^−8^	110	34	32232042 [113]
Endocytosis	4.19 × 10^−7^	142	37	15639316 [108]
TGF-beta signaling pathway	1.05 × 10^−6^	57	33	26578886 [100]
Rap1 signaling pathway	3.20 × 10^−6^	143	36	12819788 [115]
Focal adhesion	9.32 × 10^−6^	141	37	17215111 [81]
Neurotrophin signaling pathway	1.16 × 10^−5^	88	36	22654716 [99]
PI3K-Akt signaling pathway	1.45 × 10^−5^	216	37	33258115 [97]
ECM-receptor interaction	1.93 × 10^−5^	54	33	25410365 [79]
Ras signaling pathway	1.93 × 10^−5^	142	36	28374012 [106]
Phosphatidylinositol signaling system	2.28 × 10^−5^	59	35	28847278 [77]
Adrenergic signaling in cardiomyocytes	2.28 × 10^−5^	97	36	24001898 [85]
Acute myeloid leukemia	2.53 × 10^−5^	45	37	25762156 [93]
Mucin type O-Glycan biosynthesis	2.54 × 10^−5^	20	26	33218200 [89]
MAPK signaling pathway	2.54 × 10^−5^	168	37	12566928 [90]
Estrogen signaling pathway	6.29 × 10^−5^	67	36	32297302 [101]
Regulation of actin cytoskeleton	8.73 × 10^−5^	141	37	21276817 [76]
Wnt signaling pathway	0.000213	96	33	31191253 [91]
AMPK signaling pathway	0.000262	86	35	30776001 [110]
Colorectal cancer	0.000271	47	34	30323761 [80]
mTOR signaling pathway	0.000283	47	37	22202101 [94]
Oxytocin signaling pathway	0.000283	103	37	30990880 [92]
Prolactin signaling pathway	0.000645	49	37	34126620 [109]
FoxO signaling pathway	0.001453	90	36	29149835 [83]
Hepatitis B	0.001453	90	36	34398003 [102]
cGMP-PKG signaling pathway	0.001614	108	36	32715279 [111]
Tight junction	0.00311	87	36	30770921 [104]
Long-term potentiation	0.004575	48	34	27377368 [107]
Sphingolipid signaling pathway	0.006623	75	36	20571935 [88]
T cell receptor signaling pathway	0.006678	69	36	23534386 [98]
Insulin signaling pathway	0.008818	90	37	31275108 [112]
Protein processing in endoplasmic reticulum	0.009068	102	35	24832819 [87]
Gap junction	0.012445	58	35	33117125 [95]
Inositol phosphate metabolism	0.016986	42	32	15746379 [72]
Inflammatory mediator regulation of TRP channels	0.018347	64	33	32351395 [105]
Platelet activation	0.018347	80	36	9561982 [86]
Glycosaminoglycan biosynthesis—heparan sulfate/heparin	0.02388	16	24	25157361 [74]
cAMP signaling pathway	0.024452	121	36	10556645 [84]
Type II diabetes mellitus	0.031105	33	34	24526623 [82]
p53 signaling pathway	0.03515	46	35	22042001 [73]
Fc gamma R-mediated phagocytosis	0.040459	59	36	31901293 [114]

## Data Availability

For preparation of the manuscript, the publicly available database MiRTarBase was used (https://mirtarbase.cuhk.edu.cn/~miRTarBase/miRTarBase_2019/php/index.php), accessed on 21 September 2021. Cytoscape version 3.7.2, an open source platform for network visualization was used (https://cytoscape.org) (accessed on 24 September 2021). STRING was used for protein-protein interaction analysis, accessed on 24 September 2021 (https://string-db.org/). mirPath v.3 was used for miRNA enrichment analysis, accessed on 2 November 2021 (http://snf-515788.vm.okeanos.grnet.gr/). All the data are presented within the article and in supplementary material.

## Supplementary Materials

The following are available online at https://www.mdpi.com/article/10.3390/jpm11121275/s1, Table S1: Complete table of MTI data, Table S2: Complete table of enriched KEGG pathways.
